# Grazing cattle, well-managed or not, is unlikely to increase soil carbon sequestration

**DOI:** 10.1073/pnas.2203408119

**Published:** 2022-07-06

**Authors:** Kate Lajtha, Lucas Silva

**Affiliations:** ^a^Department of Crop and Soil Science, Oregon State University, Corvallis, OR 97330;; ^b^Environmental Studies Program, University of Oregon, Eugene, OR 97405

In PNAS, Rui et al. (1) present a valuable long-term dataset that, we believe, does not support the authors’ conclusion that “rotationally grazed pasture management has the potential to increase persistent soil C on Mollisols, highlighting the key role of well-managed grasslands in climate-smart agriculture.”

## Our Concerns Are as Follows

**Fig. 1.**
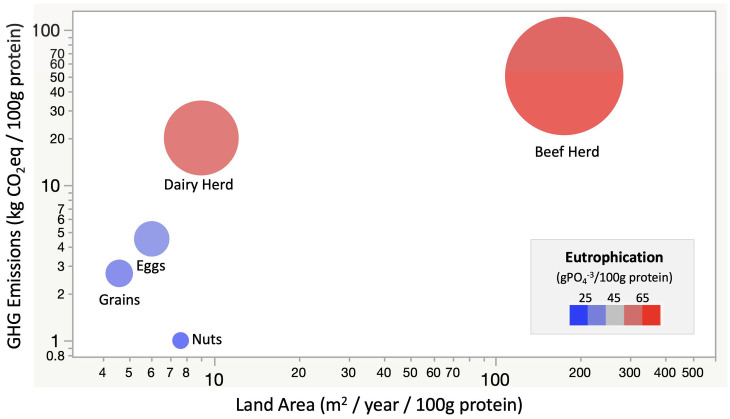
Land area (*x* axis) and greenhouse gas emissions (*y* axis) normalized to per mass of protein yield for different agricultural land uses. Circle size = CO_2_ equivalents per 100 g protein. Color gradient = nutrient requirement per 100 g protein. Average values adapted from ref. 2.

First, the authors compared grazing to crop production on an area basis, i.e., C storage per hectare. Given that both cattle grazing and crop production aim at producing food, the comparison should be on either a protein or calorie basis. Many studies estimate that beef production requires one to two orders of magnitude more land than row crops per 100 g protein (2) (Fig. 1). Therefore, a dietary shift from animal- to plant-based foods could reduce >60% of C emissions from direct agricultural production while sequestering ∼14 y of global agricultural emissions if the spared land was restored to natural vegetation (3), with associated ecosystem service benefits such as biodiversity and pollinator protection.

Second, as the authors acknowledge in this study, animals were seasonally removed from the field to be fed in enclosures for more than half of the year, presumably using food grown elsewhere, but the C footprint of that feed was not factored into their multimanagement comparison. While 86% of global livestock feed consists of materials that we cannot digest as humans, 32% of overall global grain production in 2010 was used to feed livestock, and livestock consume one-third of global cereal production (4). Thus, any calculation of C benefits/costs of cattle must include soil C effects of grain production used to feed cows.

Third, any analysis of the benefit of grazing claiming that it would “increase persistent soil C” must use an appropriate control, not only for soil C concentration but also for C residence time. We argue that the appropriate comparison for improved grazing management is not to poorly managed land excluded from native grazers (5) but rather to land grazed only by native grazers, in which proper grazing management might be variable but relatively soil-C-neutral (6). Moreover, a quantitative understanding of C persistence would require determination of C input turnover or stabilization, achievable using molecular biomarkers or radiocarbon dating (7, 8), which was not determined in Rui et al.’s experiment (1).

Finally, it has been effectively argued elsewhere that grass-based ruminants on marginal land not suitable for crop production will produce human available protein more efficiently than can food crops (9). However, other ruminant feedstocks need careful consideration; for example, using residues of crops grown for human consumption as livestock feed might further deplete soil C (10). Finally, Rui et al. (1) do not consider that the full impact of grazing on climate would be much higher than for crops due to significant methane and manure emissions (Fig. 1). Certainly, the arguments regarding the sustainability of animal versus plant food production are complex, but grazing cattle, well-managed or not, has no role in enhanced C sequestration.
